# Vadose zone infiltration and its implication for groundwater contamination risk assessment in Siloam village, Limpopo province, South Africa

**DOI:** 10.4102/jamba.v11i2.682

**Published:** 2019-07-02

**Authors:** Ivo Arrey, John O. Odiyo, Rachel Makungo, Milton Kataka

**Affiliations:** 1 Department of Hydrology and Water Resources, School of Environmental Sciences, University of Venda, Thohoyandou, South Africa; 2 Department of Mining and Environmental Geology, School of Environmental Sciences, University of Venda, Thohoyandou, South Africa

**Keywords:** Groundwater Vulnerability, Infiltration, Modelling, Siloam Village, Vadose Zone

## Abstract

Risk assessment methods and approaches are useful for environmental planning and decision-making when dealing with risk identification and reduction in a changing global context. This is particularly true for arid and semi-arid regions, such as Siloam village, Limpopo province, South Africa, where it is a common practice to apply fertilisers to the soil during planting season for increasing crop yield. Estimates of vadose zone soil moisture fluxes were used to determine the likelihood of applied agricultural fertilisers to reach the groundwater table. This study combines field observations in the study area and a one-dimensional numerical model to explore the moisture fluxes and their implications for contaminant transport in the vadose zone. Model simulations revealed a lag time of 117 days at topsoil and 913 days beyond the root zone for deep percolation of soluble non-reactive inorganic and organic additives to reach the groundwater table. Preliminary results of this study suggest that the vadose zone is permeable and the groundwater is vulnerable to contamination within the evaluated time scale. Given that disaster risks are inevitable, reasonable methods for control and mitigation of agricultural impacts at this site are highly recommended.

## Introduction

Groundwater protection is receiving significant attention because of population increase in addition to challenges in economically developing surface water supplies. This condition is more severe in arid and semi-arid regions where surface water is quite scarce. Moreover, water pollution, flood disasters and ageing water supply infrastructure accentuate the need for disaster planning and management. Because groundwater is a ubiquitous resource, this study is aimed at assessing the risk and vulnerability associated with applied agricultural contaminants to the groundwater resource in the area. Transport of contaminants in the subsurface is closely linked with hydrologic fluxes in soils and rocks making up the vadose zone. Concerns for such contaminants reaching the groundwater table and contaminating groundwater arise, although the severity is controlled by natural attenuation processes in the vadose zone. Hence, any attempt to assess and quantify subsurface contaminant transport should normally begin by evaluating infiltration and percolation through the vadose zone, which is the main focus of this study (Šimůnek et al. 2006).

The concept of groundwater vulnerability is a useful tool for risk assessment, environmental planning and decision-making when dealing with disaster management and has been described by some authors as an amorphous concept and not a measurable property. In other words, it is the tendency or likelihood that contamination will occur based on aspects such as infiltration time of water carrying contaminants, proportion of contaminants reaching the groundwater and contaminant attenuation capacity of the media through which flow takes place (Karamouz et al. 2011). Groundwater vulnerability has been categorised into intrinsic and specific vulnerability (Vrba & Zaporozec 1994). According to specialists of the COST Action 620, intrinsic vulnerability is one that is generated by human activities taking into consideration the inherent geologic, hydrologic and hydrogeologic characteristics of an area, while specific vulnerability refers to groundwater vulnerability, for example, a particular contaminant or group of contaminants (Daly et al. 2002).

Various approaches for predicting groundwater vulnerability have been suggested and are subdivided depending on the approach. Barber et al. (1993) classified them into empirical, deterministic, probabilistic and stochastic methods. Empirical methods use various physiographic attributes such as soil, geology, topography and depth to groundwater table, while deterministic approaches use simplified forms of analytical algorithms to get, for example, a Leaching Potential Index (LPI). Probabilistic and stochastic methods use numerical solutions to mathematical equations. All these methods could be grouped into three major categories, namely, overlay and index methods, process-based methods and statistical methods (Anthony et al. 1998). Process-based methods are powerful in assessing groundwater vulnerability by using deterministic approaches, for example, to estimate time of travel, contaminant concentrations and the duration of contamination to map out areas of high and low vulnerability. Examples of such process-based methods that use only the unsaturated zone include HYDRUS and Surface to Aquifer Advection Time (SAAT), while others including the saturated zone are MIKE SHE (Système Hydrologique Européen) and SWAT. Unfortunately, these process-based methods are data intensive and can only be used at local scales, rather than at regional scales, to determine capture zones for municipal wells (Liggett & Talwar 1999). Perhaps the most widely used vulnerability assessment method is the overlay and index method, such as DRASTIC (Aller et al. 1987), which uses a subjective point rating system for various hydrologic factors based on professional expertise of the developer (Schlosser et al. 2002). Statistical methods range from simple summary of descriptive statistics of concentrations of target contaminants to complex regression analyses, which employ the effects of several predictor variables (Focazio et al. 2002).

As this study employs a process-based method to assess vulnerability to the groundwater table, emphasis will be given on physically based methods. In that regard, a quantitative description of a pollution event is based on the following three factors: contaminant transfer time from hazard zone to groundwater table, contaminant duration in groundwater and the level of contaminant concentration reached in groundwater (Popescu et al. 2008). As such, an understanding of the fundamental hydrological processes specific to an area is needed before conducting groundwater vulnerability assessments. An approach based on the hazard–pathway–target model that considers a hierarchical process beginning with intrinsic vulnerability, specific vulnerability and, finally, risk assessment was suggested by Brouyére et al. (2001), which can distinguish between groundwater ‘resource vulnerability’ and ‘source vulnerability’. To the best of our knowledge, studies on solute migration in the vadose zone have focused mainly on three factors: displacement of solutes by the percolating water (Amiaz et al. 2011; Sardin et al. 1991; Simunek et al. 1998; Warrick, Biggar & Nielsen 1971), evaporation responsible for solute accumulation in the sediment profile (Nativ et al. 1995; Scanlon 1992) and geochemical interactions with sediment minerals (Bertolo, Hirata & Sracek 2006).

Modern agricultural practice applies fertilisers to the soil during planting season to increase crop yield. These fertilisers usually contain nitrate () and phosphate () chemical compounds, which, if the former compound is available beyond acceptable levels in drinking water, have been noted to cause health risks to users, such as methaemoglobinemia (blue baby syndrome) that is associated with higher-than-normal levels of methaemoglobin rather than haemoglobin (Majumdar & Gupta 2000). This practice on sites with unconfined and fractured aquifer systems poses a threat to the groundwater resource by infiltrating the groundwater table and contaminates groundwater. Concentration levels of contaminated groundwater depend on the type of contaminant and other natural attenuation processes for timescales of decades to centuries (Cozzarelli et al. 2011; Christensen et al. 2002; Milosevic et al. 2012). This study aims to evaluate intrinsic groundwater vulnerability using a physically based approach where only the transfer time from hazard zone to groundwater table is considered.

## Methodology

MIKE SHE Hydrologic Modeling Software package was used to simulate the flow to a depth of 80 cm in the vadose zone in an area dominated by residential houses and subsistence maize gardens. The MIKE SHE model is able to integrate all the hydrogeological processes involved during evaluation of water resources. It is a deterministic finite difference representation and solution of the theoretical partial differential equations describing mass and energy balance including verified empirical relations. The water movement module (MIKE SHE WM) is the major part of the model and combines different process-oriented components, each describing the main processes in the individual parts of the hydrological cycle. It also comprises modules such as the advection–dispersion or geochemical modules, which were not considered in this study because of lack of data. Details of the MIKE SHE model process flow and requirement can be found in MIKE SHE user technical manual (DHI 2007). In the methodology, it was assumed that the contaminants are soluble, non-reactive and are transported by the infiltrating water.

For a one-dimensional vertical flow, the driving force for transport of water in the vadose zone is governed by Richards’ equation with boundary conditions given in Equation 1.

$$\left\{ \begin{array}{l} \frac{\partial \theta }{\partial t}=\frac{\partial }{\partial z}\left\{K(\theta )\frac{\partial h}{\partial z}\right\}+\frac{\partial K(\theta )}{\partial z}-S(z)\\ \;{h(z,t)|}_{t=0}=h_{0}(z)\\ \;{h(z,t)|}_{z=L}=h_{b}(t) \end{array}\right.$$ [Eqn 1]

where *θ* = volumetric water content (L^3^ L^−3^), *S* = source/sink term (L^−1^), *h*_0_ = top pressure (L) *K* = hydraulic conductivity (LT^−1^), *h*_*b*_ = bottom pressure (L), *t* = time (T), *z* = vertical coordinate (L) and *L* = model depth (L).

The implicit scheme for an interior node of vertical flow yields Equation 2:

$$q_{J+\frac{1}{2}}^{n+1}=-K_{J+1/2}^{n+1/2}\left[\frac{\left(\psi_{J+1}^{n+1}-\psi_{J}^{n+1}\right)}{\Delta Z_{J+1}+1}+1\right]$$ [Eqn 2]

where *ψ* = pressure component, *J* = subscript referring to spatial increment while *n* + 1 = superscript is the time increment.

Richards’ equation requires two functions: one for the pressure head as a function of saturation and the other for the hydraulic conductivity, also as a function of saturation. Evapotranspiration (ET) acts as a water sink in the upper soil layer and root zone portion of the unsaturated zone. MIKE SHE calculates the unsaturated flow using a fully implicit finite difference solution (Refsgaard & Storm 1995). For each time step, the upper boundary condition is either a constant flux (the rainfall rate at the ground surface) or a constant head (the level of ponded water on the ground surface). In most cases, the lower boundary is a pressure boundary determined by the water table.

**FIGURE 1:**
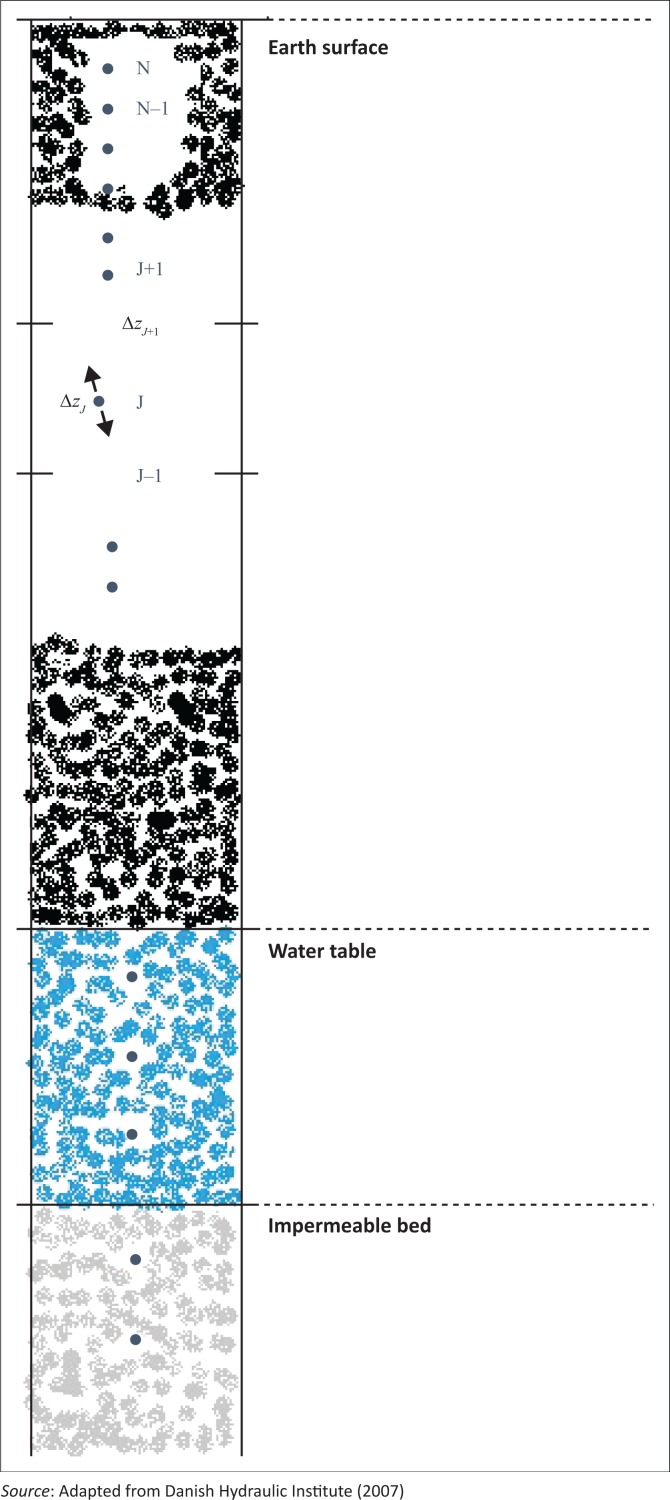
Vertical discretisation of a soil profile.

MIKE SHE includes an iterative coupling procedure between the unsaturated and saturated zones to compute the correct soil moisture and the water table dynamics in the lower part of the soil profile. Particularly in this part of the model, it is important to account for the variable specific yield above the water table, as the specific yield depends on the actual soil moisture profile and availability of that water. Flow is computed in discrete time steps for each node, as shown in Figure 1, along the specified soil profile.

### Description of study area

**FIGURE 2:**
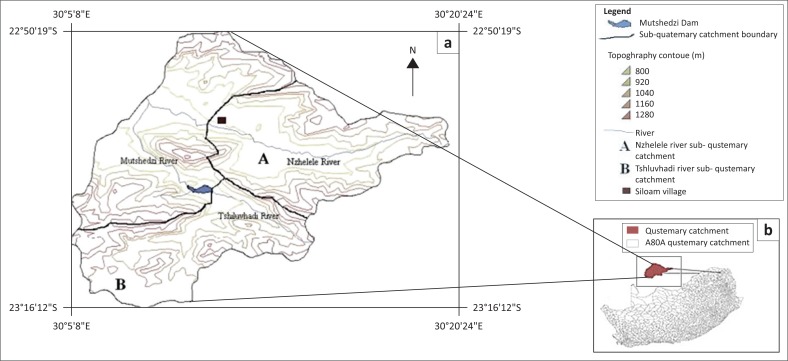
Map of (a) Siloam village in Nzhelele catchment, Limpopo, (b) South Africa.

The study area, about 24 km^2^, is located between latitudes 22°53′15.8″ S and 22°54′5″ S and longitudes 30°11′10.2″ E and 30°11′23.5″ E and falls under a delineated portion of the quaternary catchment A80A of the Nzhelele River catchment (Figure 2). It is found in the northern region of Limpopo province of South Africa and falls within the Nzhelele geological formation, which is part of the Soutpansberg Group rocks. This formation represents the topmost unit of the Soutpansberg group and consists of a 400-m-thick volcanic assemblage at the base, overlain by red argillaceous and arenaceous sediments (Brandl 1999). Located on the leeward side of the Soutpansberg Mountains, it has an average rainfall of 350 mm – 400 mm per annum and an evaporation of 1300 mm – 1400 mm per annum, making ET to sometimes exceed precipitation. Rainfall is predominant during the summer season that spans from October to March and the area has a semi-arid climate, with a mean annual temperature of 27°C.

The delineated area has a topographic range between 800 m and 860 m above sea level (m.a.s.l.). The vegetation cover is made up of patches of shrubs and grass, although there exist spontaneous places with fallow land which is periodically covered with maize crop during planting season. The area of study was delineated based on the monitoring perimeter of the catchment and the topographic gradient.

### MIKE SHE model

**FIGURE 3:**
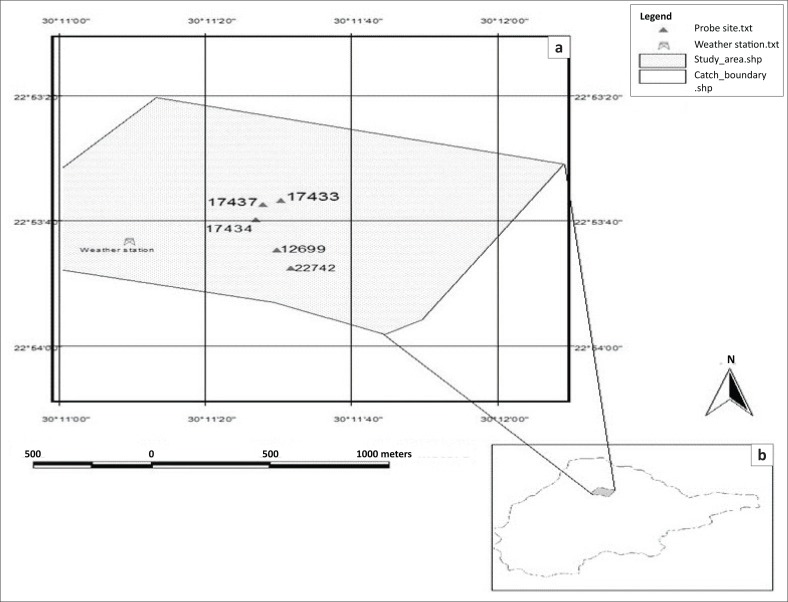
(a & b) Map showing the Nzhelele quaternary catchment monitoring area.

The MIKE SHE model was used to simulate flow in the vadose zone. Hydrometeorological data for a period of 7 months (13 January 2012 – 13 July 2012) were obtained from the University of Venda’s meteorological station in Siloam village. The meteorological station is equipped with a Davis Vantage Pro2 recorder, which is about 525 m from soil moisture monitoring probes (Figure 3). Soil moisture data were collected from three DFM neutron probe sites (17 433, 17 434 and 17 437), which record soil moisture at depths of 10 cm, 20 cm, 30 cm, 40 cm, 60 cm and 80 cm. Soil samples were collected using soil auger to determine the texture at depth increments of 0 cm–30 cm, 30 cm–60 cm and 60 cm–90 cm. Potential ET was estimated at an hourly scale using the Penman–Monteith method. This together with precipitation (P) served as the model’s upper boundary conditions. The hourly timescale was chosen so as to give a good picture of the reality as ET can vary substantially during day and night. The Soil Water Retention Curve (SWRC) and the Hydraulic Conductivity Curve (HCC) were described using the Van Genuchten (1980) function, with initial parameters shown in Table 1 obtained from the UNSODA Database (Leij et al. 1996).

**TABLE 1:** Model parameter specification for soil water retention curve and the hydraulic conductivity curve.

Parameter	Initial value	Lower bound	Upper bound
*K_s_*	3.71 × 10^−3^	1 × 10^−6^	0.01
*α*	0.124	0.1	0.5
*θ_s_*	0.85	0.41	0.9
*θ_r_*	0.067	0.057	0.08
*n*	2.28	2.22	2.68

*Source:* Leij et al. 1996

The Rooting Depth (RD) was determined from the soil profile of a 55-cm-deep pit in the study area. Preliminary sensitivity analysis was carried out prior to model calibration, and the default values of C1, C2, C3, C_int_ and A_root_ were used for calculating the actual ET (Kristensen & Jensen 1975). A digital elevation model (DEM) was generated from a digitised 1:391 478 topographical map. The model domain was set discretised into a grid size of 184 rows and 200 columns, NX and NY, respectively, with a cell size of 46 m. For time step control, an initial time step of 1 h and a maximum allowed unsaturated zone (UZ) time step of 2 h were used and modified according to the model requirements.

The model lower boundary was specified as the depth to the groundwater table determined using a Vertical Electrical Sounding Method (VES) in the area and ranged between 10.4 m and 31.6 m. This gave an average value of 21 m of the depth to groundwater in the model domain overlain by argillaceous and arenaceous sediments. Sensitivity analysis was then conducted to determine sensitive and insensitive model parameters, which served as guidelines for those parameters that needed greater attention. During the calibration, the soil hydraulic properties were found to be the most sensitive parameters in the model and were thus subjected to auto-calibration in the MIKE SHE AUTOCAL module. Model performance was evaluated based on the comparisons between the simulated and observed soil moisture. Four quantitative criteria were utilised: coefficient of correlation (R), coefficient of determination (R^2^), Nash–Sutcliffe efficiency (NSE) and root mean square error (RMSE).

### Computation of travel time to groundwater table

Using the depth to the groundwater table as target, travel time (T) in days to target at depth of 21 m was calculated using Equation 3.

$$\text{T}=\frac{\text{Distance}(\text{s})}{\text{Infiltration rate}(\emptyset )}$$ [Eqn 3]

where s = depth to groundwater (L) and Ø = infiltration rate (LT^−1^).

### Ethical consideration

This article followed all ethical standards for a research without direct contact with human or animal subjects.

## Results and discussion

A study has been conducted to evaluate intrinsic groundwater vulnerability in a delineated area of Siloam village using a physically based approach. Initially, simulations with the model parameters obtained from the UNSODA database prior to calibration yielded a not-so-good fit between the simulated and the observed soil moisture data. The MIKE SHE AUTOCAL module was then used to improve the hydraulic parameter estimates. Through inversion modelling and using the observed soil moisture for the objective function, *a priori* soil hydraulic parameters in the Van Genuchten function were optimised. The reference and optimised parameters are shown in Table 2. The optimised MIKE SHE hydrological simulation model was then used to evaluate the vulnerability of groundwater to contamination using reasonably calibrated hydraulic parameters.

**TABLE 2:** Reference and optimised model parameters.

Parameter	Initial value	Optimised value
*K_s_*	3.71 × 10^−3^	0.352 × 10^−2^
*α*	0.124	0.464
*θ_s_*	0.85	0.895
*θ_r_*	0.067	0.756 × 10^−1^
*n*	2.28	2.33

**FIGURE 4:**
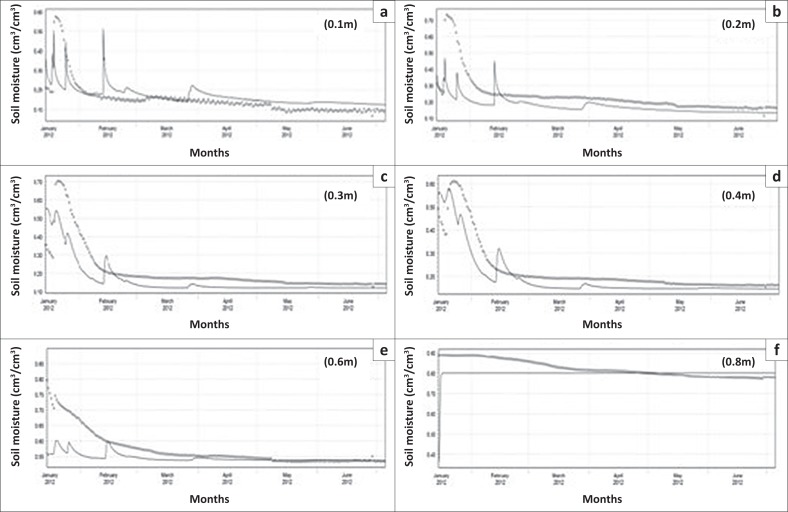
(a–f) Observed (dotted lines) versus simulated (solid line) soil moisture for probe 17434.

A granulometric analysis of the study by Arrey (2013) in the same study area indicated that the soil types at these sites are clayey and loamy sand soils. As the monitoring sites received the same amount of rainfall and have practically the same soil textural distribution, the evaluation of groundwater vulnerability was done using only probe 17 434 (Figure 3). The soil moisture results presented in the form of a graph in Figure 4 show an error difference of 0.17 cm^3^/cm^3^ between the simulated and observed values at the 0.1 m depth during the start of the simulation period, which dropped to 0.01 cm^3^/cm^3^ at the end of simulation. At 0.2 m depth, the error difference at the beginning of simulation reduced to 0.09 cm^3^/cm^3^ and dropped to 0.01 cm^3^/cm^3^ at the end of simulation like the previous depth of 0.1 m, making it a better fit to the observed data compared to the previous depth.

At 0.3 m depth, the error difference at the start of simulation was 0.28 cm^3^/cm^3^, while at the end it was 0.04 cm^3^/cm^3^, showing a slight increase in error difference than in the previous two depths. Further down at the 0.4 m depth, an error difference of 0.12 cm^3^/cm^3^ was obtained at the start and it ended with an error difference of 0.02 cm^3^/cm^3^. At 0.6 m depth, an error difference of 0.09 cm^3^/cm^3^ was obtained at the start of simulation and it ended with a 0.01 cm^3^/cm^3^ error difference between the simulated and the observed data. At 0.8 m depth, an error difference of 0.08 cm^3^/cm^3^ was obtained at the start and it ended with an error difference of 0.01 cm^3^/cm^3^. These results indicate a reduction in residual error between the simulated and the observed data. During calibration, the simulated soil moisture data at various observation nodes in the modelling generally matched the observed data, with marginal difference at the start of the simulations.

The highly non-linear nature of the soil hydraulic properties across the different soil materials making up the vadose zone could possibly account for the differences in observed and simulated soil moisture fluxes down the soil profile. This is typical with swelling argillaceous material whose response to hysteretic flow was not accounted for in the MIKE SHE model. Table 3 shows the computed statistical values for the quantification of the model performance. The RMSE values obtained fall within the acceptable range as they were close to zero (Moriasi et al. 2007). The coefficient of determination and correlation coefficient had, on average, high values, indicating less error variance on majority of the depths (Van Liew, Arnold & Garbrecht 2003). Nash–Sutcliffe values greater than or equal to 0.6 are considered satisfactory, while one is the optimal value. Values between 0 and 1 are generally viewed to be acceptable levels of model performance (Moriasi et al. 2007).

**TABLE 3:** Computed statistical values of model performance.

Depth (m)	ME	MAE	RMSE	STDres	R(Correlation)	Nash–Sutcliffe
0.1	−0.06747	0.071402	0.087289	0.055376	0.943721	0.687905
0.2	0.024826	0.047681	0.078786	0.074772	0.911421	0.811974
0.3	−0.00488	0.063912	0.092458	0.092329	0.846058	0.70929
0.4	0.093367	0.09904	0.120115	0.075567	0.837014	0.206931
0.6	0.003339	0.024604	0.031097	0.030917	0.728564	0.0776484
0.8	−0.00204	0.004578	0.00588	0.005517	0.793812	0.569496

Me, Mean Error; MAE, Mean Average Error; RMSE, Root Mean Square Error.

**FIGURE 5:**
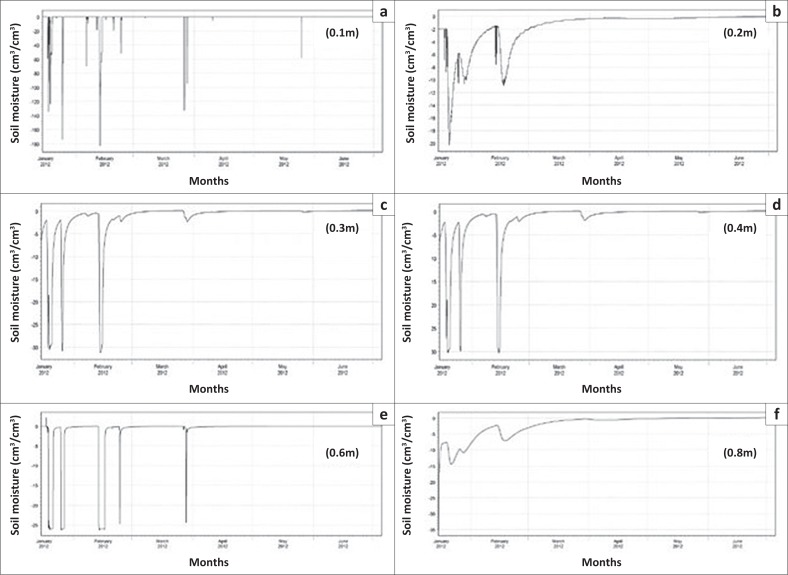
(a–f) Simulated soil moisture fluxes with depth for probe 17434.

With the fitted (optimised) hydraulic parameters, the soil moisture fluxes were simulated for 0.1 m, 0.2 m, 0.3 m, 0.4 m, 0.6 m and 0.8 m depths. The simulated soil moisture fluxes in the unsaturated zone were found to generally decrease with increasing depth throughout the soil profile as shown in Figure 5. Given that much water is available at the topsoil during precipitation, this zone had the highest recorded infiltration rates for the months of January and February, with a value of 180 mm/day at the 0.1 m depth and values as low as 23 mm/day at a depth of 0.8 m (Figure 5).

The computed travel times are presented in Table 4. There was a general increase in travel time to the groundwater table with increasing depth as a result of a drop in the infiltration rate. However, the unexpected sharp decline in infiltration rate and consequently the sharp increase in travel time at the 0.2 m depth could be attributed to the root density observed at this depth, which was taken care of in the numerical solution by the sink term in Richards’ equation [Eqn 1].

**TABLE 4:** Computed travel time for each depth increment for probe 17434.

Depth (m)	Infiltration rate (m/day)	Travel time (days)
0.1	0.18	117
0.2	2.04 × 10^−2^	1029
0.3	3.1 × 10^−2^	677
0.4	3.0 × 10^−2^	700
0.6	2.6 × 10^−2^	808
0.8	2.3 × 10^−2^	913

This intrinsic groundwater vulnerability study provides a good assessment of the travel time for soluble non-reactive solutes to be transported in the vadose zone. Vulnerability assessments using a numerical flow model, such as the MIKE SHE model, define the relative vulnerability of aquifers in terms of zonation using the average time taken by the infiltrating water to reach the aquifer at different points in space (Gogu & Dassargues 2000). Because a model is only an approximation of reality, and also because the inputs to the model are rarely exactly known with precision, the output of the model is also likely to deviate from reality. Hence, uncertainty assessments are necessary and should include uncertainty on model structure, parameter values, etc. (Refsgaard & Henriksen 2004). It is important to know how large the uncertainties in the model outputs are, particularly when the model is used for predictive purposes (Beven 2002; Heuvelink 1998). It should be noted that the attenuation coefficient, which controls transport behaviour of chemical compounds, was not considered on the assumption that the contaminant dissolved completely in solution. This method does not also consider the concentration of the contaminant and the duration of contaminant with the target, making it a unique example of intrinsic vulnerability assessment. This study uses the concept of Pressure–State–Impact (PSI) casual chain proposed by Gardin, Wojda and Brouyére (2006), which uses the generalised concept of groundwater vulnerability to evaluate any kind of stress factor that can affect any considered groundwater resource.

## Conclusion

The MIKE SHE hydrological simulation model was used to evaluate the vulnerability of groundwater to contamination using reasonably calibrated hydraulic parameters based on inversion techniques. During calibration, the simulated soil moisture data at various observation nodes in the modelling generally matched the observed data, with marginal differences. The results showed that the vadose zone fluxes are significantly controlled by the soil hydraulic properties. For a given small amount of net infiltrated precipitation, some amount of recharge takes place in the saturated zone. The contaminants dissolved in the infiltrated water will therefore reach the vadose zone.

Farming practices involving using fertilisers to increase crop yield should be properly done so that only the required amount of additives for plant uptake is used. Given that water is a universal solvent, it is expected that surface water, which encounters any contaminants such as those used in agriculture, will be transported to the saturated zone. As observed with infiltration rates as small as 0.023 m/day, contaminant-laden water will take up to 913 days to reach the groundwater table of 21 m, posing a threat to groundwater-dependent users including their ecosystems. However, this could be slower if an attenuation factor was introduced in the model, but this will not negate the threat posed to the groundwater. Therefore, careful communication between water resources managers, scientists and farmers is required for an optimal use of and hazard mitigation for groundwater resources.
